# Reducing surgical site infections

**DOI:** 10.1038/s43856-021-00055-7

**Published:** 2021-12-10

**Authors:** Katharine Barnes

**Affiliations:** Communications Medicine, https://www.nature.com/commsmed/

## Abstract

The most common complication after surgery is surgical site infection. A recent randomised controlled clinical trial published in *The Lancet* compared the rate of infections following four different wound closure regimes in low-income and middle-income countries.

Surgical site infections have been shown to be more prevalent in low-income and middle-income countries, occurring after up to 20% of operations. This led to the WHO making globally applicable recommendations for the prevention of surgical site infections. However, as there was limited data available about the challenges faced by low- or middle-income countries, a Delphi consensus process was undertaken to identify suitable interventions to test in a pragmatic, multicentre, 2 × 2 factorial, stratified randomised controlled trial, named FALCON.

The NIHR Global Research Health Unit on Global Surgery has now published the results of the FALCON trial^1^ in which the use of 2% alcoholic chlorhexidine and non-coated suture, 2% alcoholic chlorhexidine and triclosan-coated suture, 10% aqueous povidone–iodine and non-coated suture, and 10% aqueous povidone–iodine and triclosan-coated suture were compared following abdominal surgery. 5788 patients in 7 countries across Africa, Asia and America were randomly assigned to an intervention. A broad inclusion criteria was used to enable results to be obtained from any age of patient who was undergoing emergency or elective surgery for any indication. This enabled the trial to include patients and operation types that have not previously been studied in randomised controlled trials.

**Figure:**
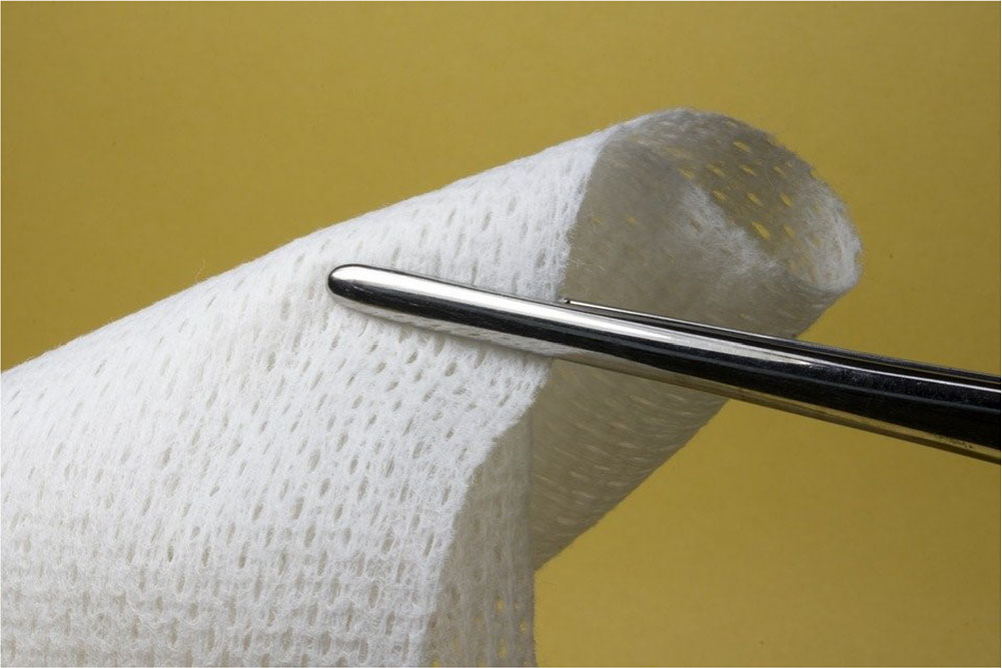
Pixabay

It was anticipated that there would be different degrees of intraoperative contamination and that these might result in differing responses to interventions. Thus, operations were assigned to two categories, clean-contaminated or contaminated/dirty, with overall infection rates shown to differ. The primary outcome of the trial was whether surgical site infections were reduced up to and at 30 day’s post-surgery, determined by telephone consultation. For both categories, all interventions gave similar reductions in surgical site infections. The secondary objectives reported in this paper assessed the effect of the trial interventions on infections at hospital discharge, need for a further operation, mortality, unplanned wound opening, lengths of hospital admissions and return to normal activities during the 30 days following the operation. Again, all interventions gave similar results.

Similar findings were seen across the globe, across age groups, and following both emergency, and elective operations. Whilst a formal health economic analysis still needs to be performed the differing price points of the interventions tested already allow some recommendations to be made. Alcoholic chlorohexidine and triclosan-coated sutures are more expensive, but were not proven to be better. Thus the authors’ immediate recommendation is that aqueous povidone–iodine and non-coated sutures be used to provide the most affordable prevention of surgical site interventions.
